# Supplementary figures and images for: Identification of quantitative trait loci (QTLs) for key cheese making phenotypes in the blue-cheese mold Penicillium roqueforti
Source: PLoS Genet. 2025 Apr 15;21(4):e1011669. doi: 10.1371/journal.pgen.1011669 (PMC12047768; doi:10.1371/journal.pgen.1011669)

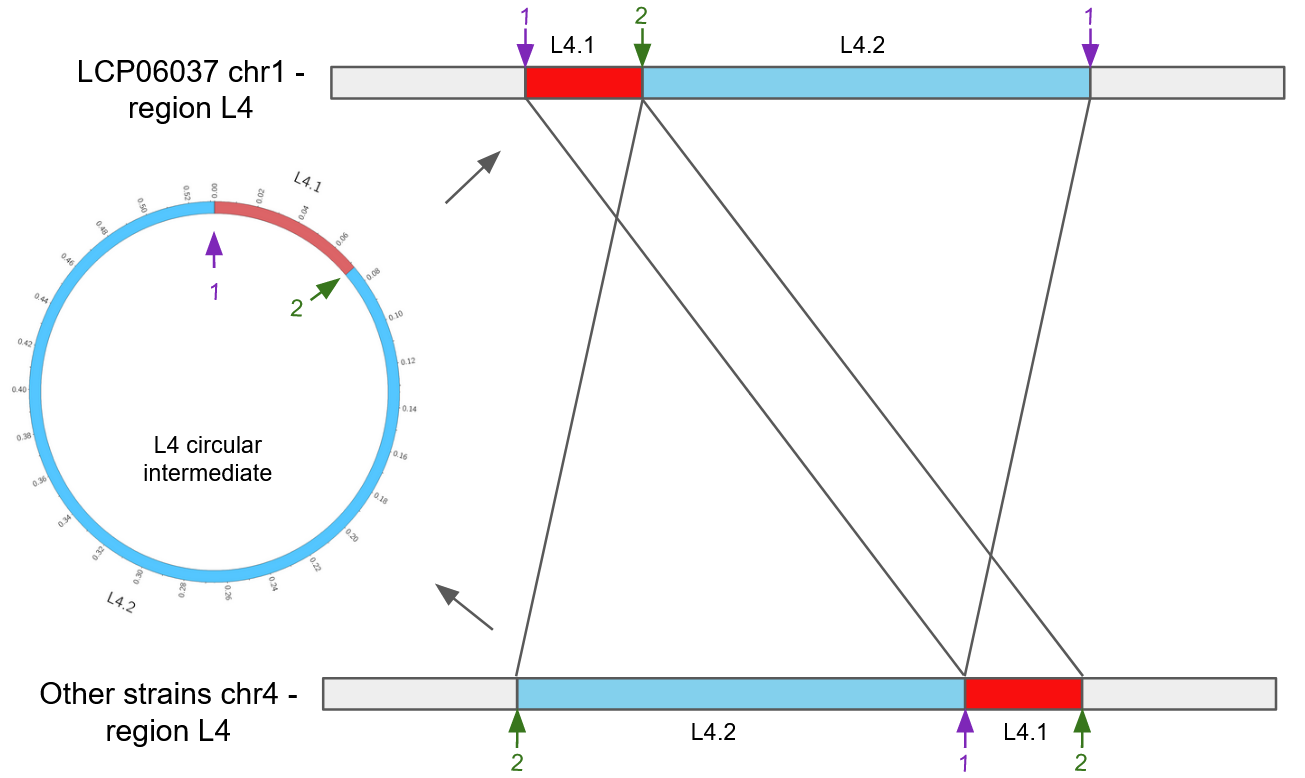

Supplement: S1 Fig — The L4 region is subdivided into two subregions, L4.1 in red and L4.2 in blue, with different relative positions, suggesting a circular intermediate for the translocation. The cut sites for the proposed circular intermediate are indicated by purple and green arrows, respectively, and numbered 1 and 2. The fig shows the synteny of the L4 translocated region present in chromosome 1 in the LCP06037 lumber parental strain and in chromosome 4 in other parents and the proposed circular intermediate. (TIF) [file pgen.1011669.s001.tif]

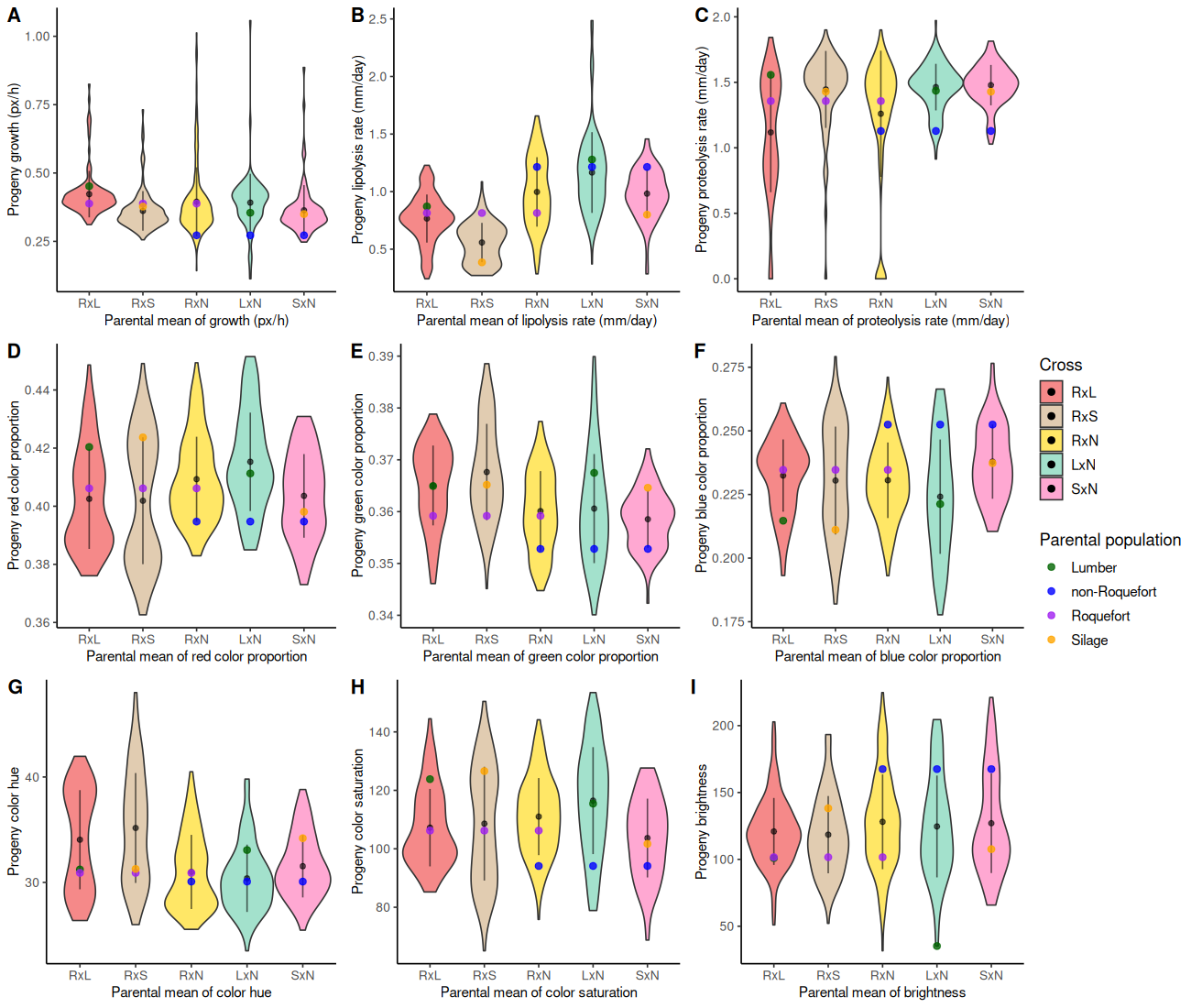

Supplement: S2 Fig — The black point and line represent the mean and standard deviation, respectively, in progenies. The coloured points represent the parental values (lumber in green, non-Roquefort in blue, Roquefort in purple, silage in orange). (TIF) [file pgen.1011669.s002.tif]

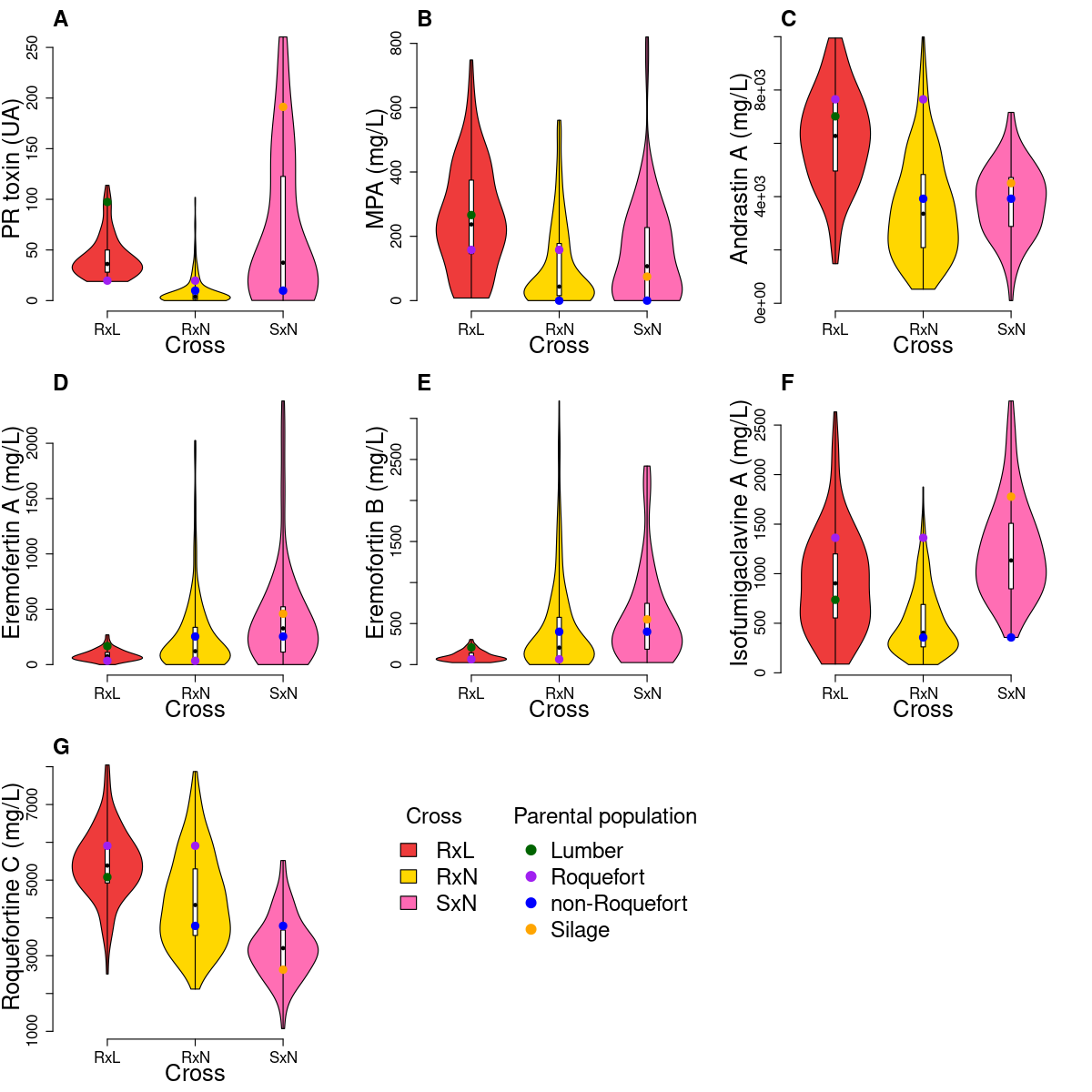

Supplement: S3 Fig — The coloured points represent the parental values (lumber in green, non-Roquefort in blue, Roquefort in purple, silage in orange). All units are in mL.L-1 except the PR toxin, which is expressed in arbitrary units. (TIF) [file pgen.1011669.s003.tif]

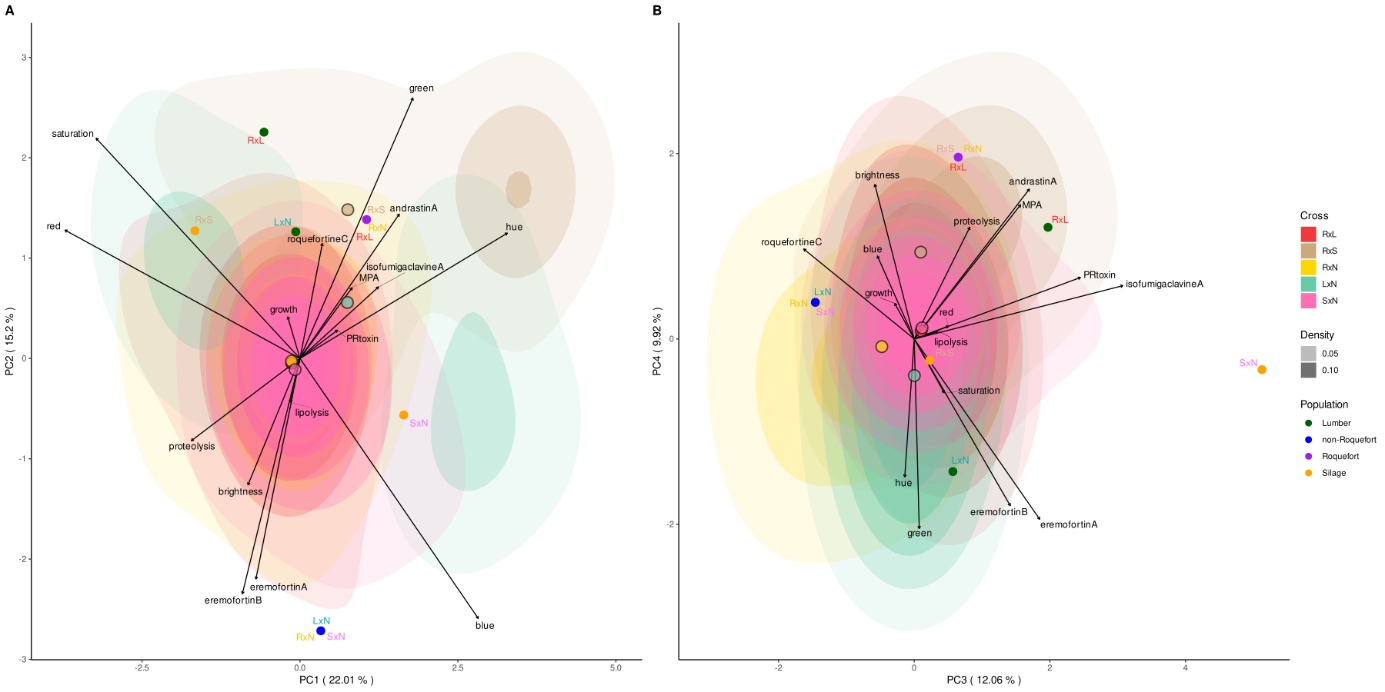

Supplement: S4 Fig — For every cross the progeny scores are displayed in a different color (LxN in red, RxL in brown, RxN in yellow, RxS in turquoise, SxN in pink) and with two density levels (from 0.05 to 0.10 in light color and above 0.10 in dark color). Large dots represent the barycentre of each progeny’s scores using the same color code (the red one is under the yellow one). The 4-fold loadings of the phenotypes are represented by the black arrows. Small dots show the scores of the parental strains from different populations (lumber in green, non-Roquefort in blue, Roquefort in purple and silage in orange) in the labels show the cross they are involved in. The percentage in the axis labels shows the total variance explained by the component. (TIF) [file pgen.1011669.s004.tif]

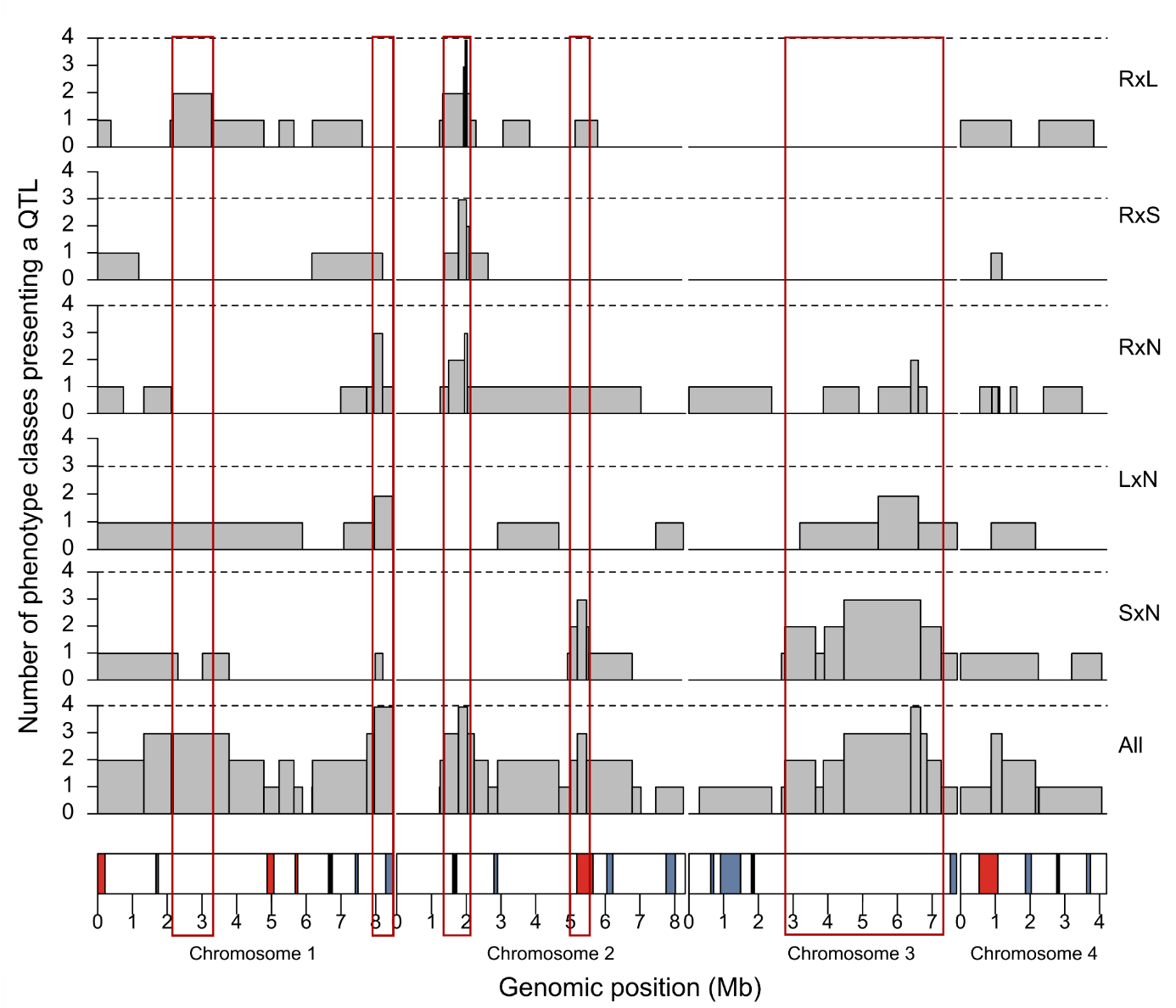

Supplement: S5 Fig — The crosses are represented one per line, with the cross ID indicated on the right, the first parent carrying the MAT1–1 mating type; the bottom line represents a pool of all crosses. The x-axis represents genomic physical positions (Mb). At the bottom, the four chromosomes of the reference genome (LCP06133) are represented by rectangles depicting genomic features: large horizontally transferred regions specific to the reference genome, in blue, and translocated regions in the reference genome, in red. The large vertical empty red rectangles indicate pleiotropic QTL regions, with effects on multiple traits in at least one cross. (TIF) [file pgen.1011669.s005.tif]

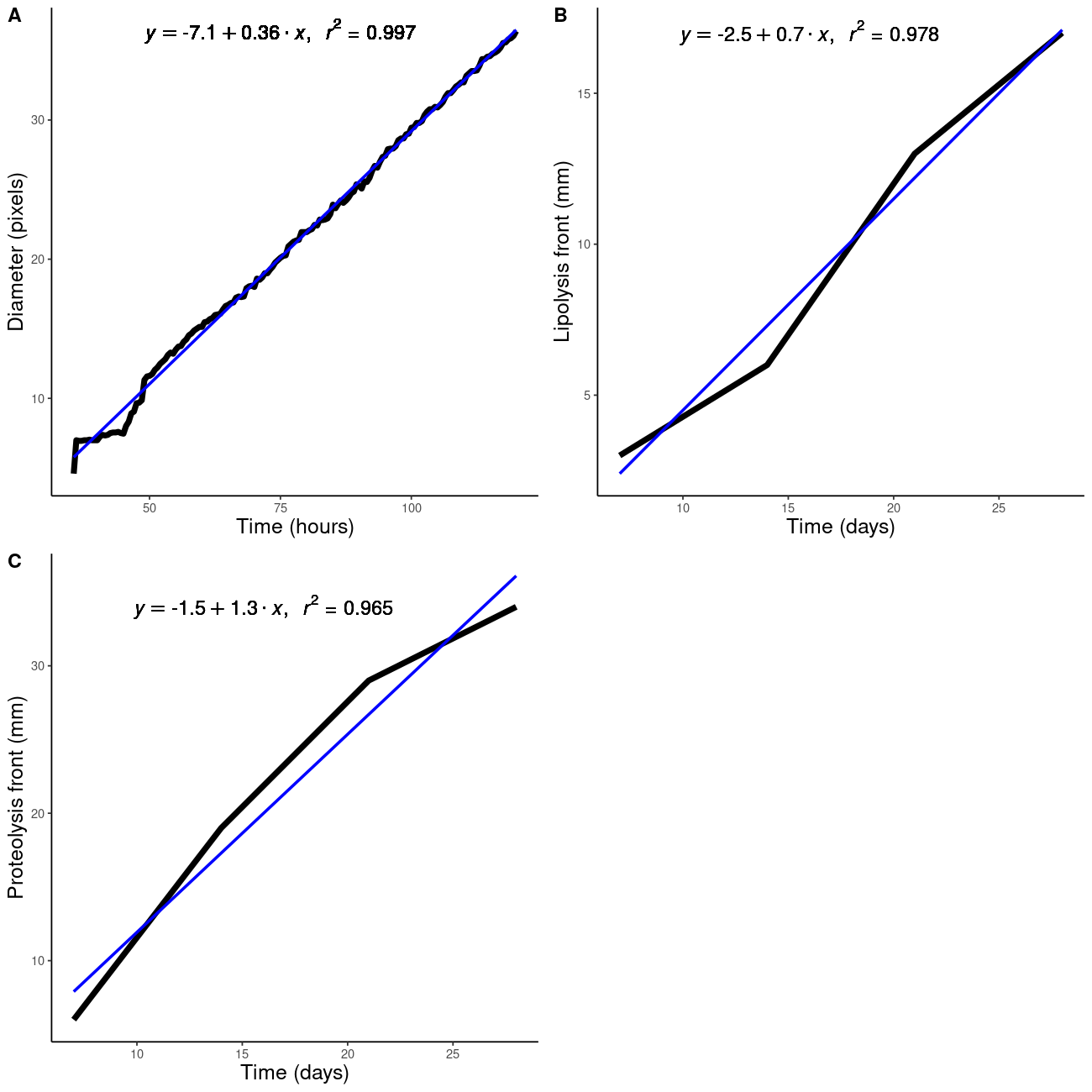

Supplement: S6 Fig — The linear regression lines are shown in blue, and their equations and coefficients of determination are given at the top. (TIF) [file pgen.1011669.s006.tif]
