# Supplementary material for: Identification of quantitative trait loci (QTLs) for key cheese making phenotypes in the blue-cheese mold Penicillium roqueforti
Source: PLoS Genet. 2025 Apr 15;21(4):e1011669. doi: 10.1371/journal.pgen.1011669 (PMC12047768; doi:10.1371/journal.pgen.1011669)
Supplement: Attachment — Submitted filename: Review_&_response.docx [file pgen.1011669.s023.docx]

Dear Editor,

We thank you and the referees for again constructive and useful comments. We have revised the manuscript along the lines suggested by you and the referees, and you will find below specific answers to the referees’ comments and your comments. We have submitted a version where changes are highlighted in yellow.

Best regards

Ewen Crequer, on the behalf of all authors

Dear Dr Crequer,

Thank you very much for submitting your Research Article entitled 'Generation of diversity in the blue cheese mold Penicillium roqueforti and identification of pleiotropic QTL for key cheese-making phenotypes' to PLOS Genetics.

The manuscript was fully evaluated at the editorial level and by independent peer reviewers. The reviewers appreciated the attention to an important topic but identified some concerns that we ask you address in a revised manuscript. We therefore ask you to modify the manuscript according to the review recommendations. Your revisions should address the specific points made by each reviewer.

Required changes are:

-Shortening of both the introduction and the discussion sections, possibly combining the results and discussion sections; while doing this, it is essential to provide a broader context, referring to the existence or not of comparable studies in fungi and confronting the results of the present study with findings by others, if possible.

>>In accordance with your request, we have greatly shortened both the introduction and discussion and removed the associated references. However, after deep consideration, we have decided to keep the results separated from the discussion, as we think that this structure is easier for readers to read, understand and find what they look for, but we have deleted potential redundancies between the discussion and the result section. We also included additional references to provide more in depth comparison with previous studies.

Removed references:

Baack, E. J., Sapir, Y., Chapman, M. A., Burke, J. M., & Rieseberg, L. H. (2008). Selection on domestication traits and quantitative trait loci in crop–wild sunflower hybrids. *Molecular Ecology*, *17*(2), 666–677. <https://doi.org/10.1111/j.1365-294X.2007.03596.x>

Bernardes, J. P., Stelkens, R. B., & Greig, D. (2017). Heterosis in hybrids within and between yeast species. *Journal of Evolutionary Biology*, *30*(3), 538–548. <https://doi.org/10.1111/jeb.13023>

Buchanan, R. L., Golden, M. H., & Whiting, R. C. (1993). Differentiation of the effects of pH and lactic or acetic acid concentration on the kinetics of *Listeria monocytogenes* inactivation. *Journal of Food Protection*, *56*(6), 474–479. <https://doi.org/10.4315/0362-028X-56.6.474>

Conrado, R., Gomes, T. C., Roque, G. S. C., & De Souza, A. O. (2022). Overview of bioactive fungal secondary metabolites: Cytotoxic and antimicrobial compounds. *Antibiotics*, *11*(11), 1604. <https://doi.org/10.3390/antibiotics11111604>

Cosciani-Cunico, E., Dalzini, E., Ducoli, S., Sfameni, C., Bertasi, B., Losio, M.-N., Daminelli, P., & Varisco, G. (2015). Behaviour of *Listeria monocytogenes* and *Escherichia coli* O157:H7 during the cheese making of traditional raw-milk cheeses from Italian Alps. *Italian Journal of Food Safety*, *4*(2). <https://doi.org/10.4081/ijfs.2015.4585>

Cryer, N. C., Butler, D. R., & Wilkinson, M. J. (2005). High throughput, high resolution selection of polymorphic microsatellite loci for multiplex analysis. *Plant Methods*, *1*, 1–5. <https://doi.org/10.1186/1746-4811-1-3>

Doebley, J., Stec, A., & Hubbard, L. (1997). The evolution of apical dominance in maize. *Nature*, *386*(6624), 485–488. <https://doi.org/10.1038/386485a0>

Gladieux, P., Ropars, J., Badouin, H., Branca, A., Aguileta, G., De Vienne, D. M., Rodríguez de la Vega, R. C., Branco, S., & Giraud, T. (2014). Fungal evolutionary genomics provides insight into the mechanisms of adaptive divergence in eukaryotes. *Molecular Ecology*, *23*(4), 753–773. <https://doi.org/10.1111/mec.12631>

Jadhav, M. S. (2015). An update on important functionally characterized genes / QTLs of agronomic importance in crop plants. *Indian Research Journal of Genetics & Biotechnology*, *7*(1), 44–49.

Jakubczyk, K., Kałduńska, J., Kochman, J., & Janda, K. (2020). Chemical Profile and Antioxidant Activity of the Kombucha Beverage Derived from White, Green, Black and Red Tea. *Antioxidants*, *9*(5), 447. <https://doi.org/10.3390/antiox9050447>

Laurent, B., Moinard, M., Spataro, C., Chéreau, S., Zehraoui, E., Blanc, R., Lasserre, P., Ponts, N., & Foulongne-Oriol, M. (2021). QTL mapping in *Fusarium graminearum* identified an allele of FgVe1 involved in reduced aggressiveness. *Fungal Genetics and Biology*, *153*, 103566. https://doi.org/10.1016/j.fgb.2021.103566

O’Gorman, C. M., Fuller, H. T., & Dyer, P. S. (2009). Discovery of a sexual cycle in the opportunistic fungal pathogen *Aspergillus fumigatus*. *Nature*, *457*, 471–475. https://doi.org/10.1038/nature07528

Petrizzelli, M., De Vienne, D., & Dillmann, C. (2019). Decoupling the Variances of Heterosis and Inbreeding Effects Is Evidenced in Yeast’s Life-History and Proteomic Traits. *Genetics*, *211*(2), 741–756. <https://doi.org/10.1534/genetics.118.301635>

Vernozy-Rozand, C., Mazuy-Cruchaudet, C., Bavai, C., Montet, M. P., Bonin, V., Dernburg, A., & Richard, Y. (2005). Growth and survival of *Escherichia coli* O157:H7 during the manufacture and ripening of raw goat milk lactic cheeses. *International Journal of Food Microbiology*, *105*(1), 83–88. <https://doi.org/10.1016/j.ijfoodmicro.2005.05.005>

Yao, J., Xu, J., Cornille, A., Tomes, S., Karunairetnam, S., Luo, Z., Bassett, H., Whitworth, C., Rees‐George, J., Ranatunga, C., Snirc, A., Crowhurst, R., De Silva, N., Warren, B., Deng, C., Kumar, S., Chagné, D., Bus, V. G. M., Volz, R. K., … Gleave, A. P. (2015). A *micro RNA* allele that emerged prior to apple domestication may underlie fruit size evolution. *The Plant Journal*, *84*(2), 417–427. https://doi.org/10.1111/tpj.13021

Additional references:

Auxier B, Debets AJM, Stanford FA, Rhodes J, Becker FM, Marquez FR, et al. The human fungal pathogen *Aspergillus fumigatus* can produce the highest known number of meiotic crossovers. PLOS Biology. 2023;21: e3002278. doi:[10.1371/journal.pbio.3002278](https://doi.org/10.1371/journal.pbio.3002278)

Braun-Galleani S, Dias JA, Coughlan AY, Ryan AP, Byrne KP, Wolfe KH. Genomic diversity and meiotic recombination among isolates of the biotech yeast *Komagataella phaffii* (*Pichia pastoris*). Microbial Cell Factories. 2019;18: 211. doi:[10.1186/s12934-019-1260-4](https://doi.org/10.1186/s12934-019-1260-4)

Chakraborty P, Pankajam AV, Lin G, Dutta A, Krishnaprasad GN, Tekkedil MM, et al. Modulating crossover frequency and interference for obligate crossovers in *Saccharomyces cerevisiae* meiosis. G3 Genes|Genomes|Genetics. 2017;7: 1511–1524. doi:[10.1534/g3.117.040071](https://doi.org/10.1534/g3.117.040071)

Gong W, Shen N, Zhang L, Bian Y, Xiao Y. Chromosome-wide characterization of intragenic crossover in shiitake mushroom, *Lentinula edodes*. J Fungi (Basel). 2021;7: 1076. doi:[10.3390/jof7121076](https://doi.org/10.3390/jof7121076)

Keele GR, Crouse WL, Kelada SNP, Valdar W. Determinants of QTL mapping power in the realized collaborative cross. G3 (Bethesda). 2019;9: 1707–1727. doi:[10.1534/g3.119.400194](https://doi.org/10.1534/g3.119.400194)

Pelé A, Falque M, Trotoux G, Eber F, Nègre S, Gilet M, et al. Amplifying recombination genome-wide and reshaping crossover landscapes in Brassicas. PLOS Genetics. 2017;13: e1006794. doi:[10.1371/journal.pgen.1006794](https://doi.org/10.1371/journal.pgen.1006794)

Keurentjes JJB, Bentsink L, Alonso-Blanco C, Hanhart CJ, Blankestijn-De Vries H, Effgen S, et al. Development of a near-isogenic line population of *Arabidopsis thaliana* and comparison of mapping power with a recombinant inbred line population. Genetics. 2007;175: 891–905. doi:[10.1534/genetics.106.066423](https://doi.org/10.1534/genetics.106.066423)

Stapley J, Feulner PGD, Johnston SE, Santure AW, Smadja CM. Variation in recombination frequency and distribution across eukaryotes: patterns and processes. Philos Trans R Soc Lond B Biol Sci. 2017;372: 20160455. doi:[10.1098/rstb.2016.0455](https://doi.org/10.1098/rstb.2016.0455)

Steinsaltz D, Dahl A, Wachter KW. On negative heritability and negative estimates of heritability. Genetics. 2020;215: 343–357. doi:[10.1534/genetics.120.303161](https://doi.org/10.1534/genetics.120.303161)

-Provide ascospore viability data

>>We do not have this information, as we only picked up germinating ascospores for the analysed progenies. For choosing fertile crosses, we only used qualitative assessment on the quantity of cleistothecia and the presence of ascospores within cleistothecia. We have clarified this point in the manuscript L178.

-Quantify diversity in the species and discuss it in context

>>Reading reviewer #2 comment, we realised that the use of the term “diversity” was ambiguous. We have clarified in the manuscript that we actually meant new allelic combinations rather than higher diversity level L50, L184, L464. Our discussion on diversity was intended to highlight the low diversity in the cheese populations, not in the whole species; we cannot compare the diversity within cheese populations and within the single progeny analysed between the two cheese populations, in part because we used GBS, only covering a part of the genome, and also because there are necessarily only two alleles in the progeny per site.

-Provide accession numbers for DNA sequence data, primer sequences and progeny genotype data in supplementary files.

>>We have added a table with currently available accession numbers, named Supplementary Table 5. Other genomic data and GBS data have been submitted, but they do not yet have accession numbers. These will be updated upon acceptance. Primer sequences are now presented in Supplementary Table 9 (previously Supplementary Table 8).

It is also important to go thoroughly into the question of why it was not possible to perform replicates for phenotype quantifications, as this was considered important for accuracy of the results.

>>Replicates provide finer estimates but, due to room and budget constraints for measuring traits, we had a trade-off between the number of offspring and the precision of estimates per offspring. Having more offspring provides much more power than having finer estimates per offspring, so we have chosen this option (see references below). We have added this explanation L761-763 in the manuscript.

Keele, G.R., et al., Determinants of QTL Mapping Power in the Realized Collaborative Cross. G3 Genes|Genomes|Genetics, 2019. 9(5): p. 1707-1727.

Keurentjes, J.J.B., et al., Development of a Near-Isogenic Line Population of Arabidopsis thaliana and Comparison of Mapping Power With a Recombinant Inbred Line Population. Genetics, 2007. 175(2): p. 891-905.

Lorenz, A.J., Resource Allocation for Maximizing Prediction Accuracy and Genetic Gain of Genomic Selection in Plant Breeding: A Simulation Experiment. G3 Genes|Genomes|Genetics, 2013. 3(3): p. 481-491.

We also strongly recommend consideration of the proposed changes to title and to the structure of the results section, which could improve readability and broaden the audience of the paper.

>>We have changed the title and made the suggested changes in the introduction, results and discussion sections.

In addition we ask that you:
1) Provide a detailed list of your responses to the review comments and a description of the changes you have made in the manuscript.
2) Upload a Striking Image with a corresponding caption to accompany your manuscript if one is available (either a new image or an existing one from within your manuscript). If this image is judged to be suitable, it may be featured on our website. Images should ideally be high resolution, eye-catching, single panel square images. For examples, please browse our [archive](https://journals.plos.org/plosgenetics/volume). If your image is from someone other than yourself, please ensure that the artist has read and agreed to the terms and conditions of the Creative Commons Attribution License. Note: we cannot publish copyrighted images.

>>We have uploaded a striking image.

We hope to receive your revised manuscript within the next 30 days. If you anticipate any delay in its return, we would ask you to let us know the expected resubmission date by email to plosgenetics@plos.org.

If present, accompanying reviewer attachments should be included with this email; please notify the journal office if any appear to be missing. They will also be available for download from the link below. You can use this link to log into the system when you are ready to submit a revised version, having first consulted our [Submission Checklist](http://journals.plos.org/plosgenetics/s/submit-now#loc-submission-checklist).

While revising your submission, please upload your figure files to the [Preflight Analysis and Conversion Engine](http://pace.apexcovantage.com/) (PACE) digital diagnostic tool. PACE helps ensure that figures meet PLOS requirements. To use PACE, you must first register as a user. Then, login and navigate to the UPLOAD tab, where you will find detailed instructions on how to use the tool. If you encounter any issues or have any questions when using PACE, please email us at figures@plos.org.

Please be aware that our [data availability policy](http://journals.plos.org/plosgenetics/s/data-availability) requires that all numerical data underlying graphs or summary statistics are included with the submission, and you will need to provide this upon resubmission if not already present. In addition, we do not permit the inclusion of phrases such as "data not shown" or "unpublished results" in manuscripts. All points should be backed up by data provided with the submission.

To enhance the reproducibility of your results, we recommend that you deposit your laboratory protocols in [protocols.io](http://protocols.io/), where a protocol can be assigned its own identifier (DOI) such that it can be cited independently in the future. Additionally, PLOS ONE offers an option to publish peer-reviewed clinical study protocols. Read more information on sharing protocols at <https://plos.org/protocols?utm_medium=editorial-email&utm_source=authorletters&utm_campaign=protocols>

Please review your reference list to ensure that it is complete and correct. If you have cited papers that have been retracted, please include the rationale for doing so in the manuscript text, or remove these references and replace them with relevant current references. Any changes to the reference list should be mentioned in the rebuttal letter that accompanies your revised manuscript. If you need to cite a retracted article, indicate the article’s retracted status in the References list and also include a citation and full reference for the retraction notice.

PLOS has incorporated [Similarity Check](http://secure-web.cisco.com/1VuhvnrIH1TlBs8zSeih6QWsjzyLfwXE1aO1zmq0U9MAbqt4kskkKWy_o-8kWRPIABf-gasjRLRJ6--2owdXwBZ-Xl-HLWDIVsvq2-2scIl1Vvxp1mDWbnxGNgxARz4SM6dAkplaOPwlRqbixojRPygjaAIz3RQ4V-iEFX8ps--WvSV3OLB8QBL5kYPr1liiE9ww8qnSPZbTGNOPxd6ayqfgBrNrwoqJ4-DGyjgtSwFr2whldNu3zDsOYFQo3wh4lpr46gJq7t6K1IZnEXixaiZDhTLS2cmzo2jnh_Ldw3BhyHTJKOZwV92wDq7zeDTUTIuBovdEkxAKZSJy14b4uobttFP3ufJehAheH7atj5DjQDoGtNaBDNS7IjLkKbr3hmjAmDoeE5aDtInWcZml3wVnZsSnhGkTs1GLGR0d5t6U/http%3A%2F%2Fwww.crossref.org%2Fcrosscheck.html), powered by iThenticate, into its journal-wide submission system in order to screen submitted content for originality before publication. Each PLOS journal undertakes screening on a proportion of submitted articles. You will be contacted if needed following the screening process.

To resubmit, you will need to go to the link below and 'Revise Submission' in the 'Submissions Needing Revision' folder.

Reviewer's Responses to Questions

**Comments to the Authors:
Please note here if the review is uploaded as an attachment.**

Reviewer #1: This is a very clearly presented study on an industrially important fungus. I enjoyed reading it but have only a few comments.

>>We thank the referee for this positive comment.

1. What is the ascospore viability/germination rate for each of the five crosses?

>>We do not have this information, as we only picked up germinating ascospores for the analysed progenies. For choosing fertile crosses, we first chose among crosses tested and proved fertile in Ropars et al. (2016, Evolution). Among the crosses retained in this first filter, we only used qualitative assessment on the quantity of cleistothecia and the presence of ascospores within cleistothecia to choose the final crosses to be analysed. We have clarified this point in the manuscript L172-174 .

Can the viability differences among crosses be attributed to specific chromosomal structural differences such as translocations and inversions? This is an important point for downstream breeding as it can impact whether certain recombinants can be generated during meiosis.

>>Indeed, the viability issues can be due to the identified translocations. We have added a sentence in the discussion L437-439 and this possibility was already mentioned in the parts on the segregation biases possibly due to inviability because of the translocation leading to the lack of some genomic regions L207.

2. I think the estimated narrow-sense heritabilities are highly problematic. The total genetic variance for a phenotype includes additive variance, dominance variance (which doesn't exist in haploids such as in the case here), and epistatic variance. The traditional "narrow-sense heritability" refers only to the contribution of additive variance to the total phenotypic variance and is always positive. I think the estimated "narrow-sense heritabilities" in the current version mostly reflect the epistatic gene interactions within each of the parental strains, not the additive effects. Otherwise, the h^2 value (at least for some traits) can't be negative.

>>Epistatic variance does not affect narrow sense heritability estimate, i.e. additive genetic variance estimate, when doing parent-offspring regression. Indeed, to do such regression, one needs to calculate the average offspring phenotype value of a cross and the average value of the parents. The offspring should comprise all combinations of alleles depending on recombination. Therefore, epistasis should influence the variance of the phenotypic values of offspring, not the average. However, we agree that our narrow sense values are in some cases problematic but what causes it is more likely segregation bias which will favor one parental allele over the other, thus modifying the average phenotypic value of the offspring. However, we cannot exclude an effect of epistasis on our estimates given the low number of crosses. We have clarified this in the manuscript and we thank the referee for this comment. L297-306.

3. Line 657: what proportion of the germinated spores were recombinants?

>>For the Roquefort x non-Roquefort cross, 1234 out of 1620 isolates (76%) appeared as recombinants based on 11 microsatellite markers. The detected non-recombinant ones had therefore been removed. We have added this information to the new manuscript version L175-176.

4. Line 824: Why can't the authors have replicates for phenotype quantifications for each progeny (and parental strains)? Replicates are crucial for accurate assessments of quantitative traits.

>>Replicates provide finer estimates but, due to constraints for measuring traits, we had a trade-off between the number of offspring and the precision of estimates. Having more offspring provides much more power than having finer estimates per offspring, so we chose to measure more offspring rather than obtaining finer estimates per offspring (see references cited above).

Reviewer #2: Understanding the genetic basis of quantitative traits is crucial for adaptation and breeding in domesticated organisms. Penicillium roqueforti, vital in blue cheese maturation, offers insights into flavor development. This species presents a low genetic diversity but the existence of different populations allows linkage mapping. By crossing cheese and non-cheese strains, the authors generated F1 progenies. In parallel, they analyzed parental genome assemblies, revealing large translocations and segregation distortions in offspring. Some regions exhibited transgressive segregation for traits relevant to cheese production. Quantitative trait loci (QTL) were identified for various traits, with pleiotropic effects observed. Several QTL corresponded to known biosynthetic gene clusters. F1 hybrids, with enhanced traits and diversity, offer promise for cheese production. This study advances understanding of rapid adaptation, uncovering convergent adaptation mechanisms.

The manuscript is pleasant to read, even if it is sometimes unnecessarily very long. The background is well presented but some parts could be shortened. The questions are clearly asked and the analyzes provide clear results, which fall within the scope of PLoS Genetics.

>>We thank the referee for the positive assessment. We have shortened the introduction and discussion and hope it is now satisfactory.

However, I have several points that need to be addressed:

Major points:

1. I think the title is not very appropriate. Performing linkage analysis systematically leads to an increase in genetic diversity in one way or another and is not an achievement in itself. Furthermore, this increase in diversity is not quantified but I will come back to it later. In the second part of the title, the adjective pleiotropic is perhaps intriguing. Are cheese-making traits more pleiotropic than others?
Ultimately this study lies in determining the genetic origin of cheese-making traits in Penicillium roqueforti. And this is it!

>>We have changed the title following the suggestion.

2. The authors talk about the genetic diversity and the fact this diversity is very low in this species. It would be nice to have a precise idea of this diversity. First, it would be interesting to have a precise idea of this diversity (Pi, Theta or % of divergence based on SNPs) and to compare it to other species of fungi and yeast. Second, regarding the isolates that are used in the context of linkage analyses, it would be interesting to have the pairwise genetic diversity between all the strains used. Finally, regarding the population mapping, it would also be essential to estimate this increase in diversity since the authors want to make it a punchline of the paper.

>>Reading your comments, we realised that the use of the term “diversity” was ambiguous. We have clarified in the manuscript that we actually meant new allelic combinations rather than higher diversity per se L41, L151, L418. Indeed, our discussion on diversity was intended to highlight the low diversity in the cheese populations, not in the whole species. We cannot compare the diversity within cheese populations and within the single progeny analyzed between the two cheese populations, in part because we used GBS, only covering a part of the genome, and also because there are necessarily only two alleles in the progeny per site.

3. What is the added value of the part focusing on recombination. Have other studies been carried out on other fungi? Is it possible to make a comparison that would bring additional interest?

>>We agree that the information on the estimates of cM/Mb are not directly relevant and difficult to compare to other species, so we have deleted these sentences. The number of crossing-overs per chromosome is more relevant and we have added some comparison with other fungal species. In eukaryotes, the mean number of crossing-overs is often around two per chromosome, with variability across crosses (Pelé *et al*, 2017), which is in agreement with our findings in *P. roqueforti*. In the few fungal species for which this has been estimated the numbers of crossing over per chromosome were as follows: between 4-10 in yeasts, such as *Saccharomyces cerevisiae* (Chakraborty *et al.*, 2017) or *Komagataella phaffii* (Braun-Galleani et al., 2019), above 20 in *Aspergillus fumigatus* (Auxier *et al.*, 2023), and mean number between 0.25 and 1.8 in the basidiomycetes *Hericium erinacum* and *Lentinula edodes* (Gong *et al.*, 2021). Thus, our estimates in *P. roqueforti* are among the lowest number of crossing over by chromosome in fungal species. We have added this information and the following references in the manuscript L476-488.

Auxier, B., Debets, A.J.M., Stanford, F.A., Rhodes, J., Becker, F.M., Marquez, F.R., Nijland, R., Dyer, P.S., Fisher, M.C., Heuvel, J. van den & Snelders, E. (2023) The human fungal pathogen *Aspergillus fumigatus* can produce the highest known number of meiotic crossovers. *PLOS Biology*, **21**, e3002278.

Braun-Galleani, S., Dias, J.A., Coughlan, A.Y., Ryan, A.P., Byrne, K.P. & Wolfe, K.H. (2019) Genomic diversity and meiotic recombination among isolates of the biotech yeast *Komagataella phaffii* (*Pichia pastoris*). *Microbial Cell Factories*, **18**, 211.

Chakraborty, P., Pankajam, A.V., Lin, G., Dutta, A., Krishnaprasad, G.N., Tekkedil, M.M., Shinohara, A., Steinmetz, L.M. & Nishant, K.T. (2017) Modulating crossover frequency and interference for obligate crossovers in *Saccharomyces cerevisiae* meiosis. *G3 Genes|Genomes|Genetics*, **7**, 1511–1524.

Gong, W., Shen, N., Zhang, L., Bian, Y. & Xiao, Y. (2021) Chromosome-wide characterization of intragenic crossover in shiitake mushroom, *Lentinula edodes*. *Journal of Fungi (Basel, Switzerland)*, **7**, 1076.

Pelé, A., Falque, M., Trotoux, G., Eber, F., Nègre, S., Gilet, M., Huteau, V., Lodé, M., Jousseaume, T., Dechaumet, S., Morice, J., Poncet, C., Coriton, O., Martin, O.C., Rousseau-Gueutin, M. & Chèvre, A.-M. (2017) Amplifying recombination genome-wide and reshaping crossover landscapes in Brassicas. *PLOS Genetics*, **13**, e1006794.

4. Concerning the pleiotropy of the traits studied, are they more pleiotropic than the traits studied elsewhere? If so, it would be good to make an exhaustive comparison!

>>We do cite other studies saying that pleiotropy has often been found in domesticated organisms, but an exhaustive comparison would be out of the scope of this study and would result in a lengthier manuscript, while referees asked to shorten it. Here are the citations in the article: Sweeney and McCouch 2007; Baach et al. 2008; Bachlava et al. 2010; Wang et al. 2010; Telias et al. 2011; Wirén and Jensen 2011; Andargie et al. 2014; Johnsson et al. 2014; Wright 2015; Wright et al. 2015; Kongjaimun et al. 2012; Kantar et al. 2017; Somta et al. 2020; Bomblies and Doebley 2006; Martìnez-Ainsworth and Tenaillon 2016.

Minor points:

1. the introduction is very long and could be shortened to go straight to the point.

>>We have shortened the introduction and hope it is now satisfactory.

2. the structure of the results section is somewhat surprising and confusing when reading. It probably needs to be restructured. For example, the paragraph “translocation between parental genomes” characterized the parental genomes. Why is it placed after phenotype distribution and heritability? The same goes for genetic maps, recombination and co. For reading, it would be better to have a genotype part, parental genomes, crosses, recombination then a trait part and finally the results of the linkage mapping

>>We have changed the order of the paragraphs in the result section as suggested. Figure and Table numbers were changed to follow the new order.

3. The discussion is redundant with the results section. In fact, results are simple and don’t need to be rephrase a second time.

>>We have shortened the discussion and deleted these repetitions.

Reviewer #3: This is an interesting manuscript showing how quantitative genetics can help to shed new lights in controlling phenotypic variation, although the QTLs intervals mapped are too large to identify the causative variants. One aspect of interest is the presence of large translocation among the lineages used in the crosses. I think this aspect is interesting and I would show it in more depth in manuscripts.

>>We thank the referee for the positive assessment and we have added information as suggested, see below.

Here some suggestions:

- what is the impact of translocation in gametes viability? Is gametes viability estimated? Large reciprocal translocations are expected to have a significant drop in gametes viability (e.g. 50%).

>>This was already mentioned in the parts on the segregation biases possibly due to inviability because of the translocation leading to missing genomic parts L435. However, to enhance clarity we have added a sentence in the discussion L437-439. We did not estimate gamete viability here, but we showed in a previous study (Ropars *et al.*, 2016) that gamete viability is low in the crosses involving cheese strains, this has been added in the sentence L438.

-are the surviving spore segregating the parental combinations of the translocated chromosomes or there are examples of CNV (e.g. aneuplodies)

>>We did not have heterozygous offspring or deletion for whole chromosomes, so there was no case of aneuploidy. We have added this information in the manuscript L200-206.

- if CNVs are detected, are these link to phenotypic variation (e.g. can they explain the transgressive phenotypes) or are they have fitness cost?

>>We found no case of aneuploidy, we have added this information in the manuscript L204.

- perhaps would also be interesting to include a more in depth comparison of the distribution of the recombination maps between the different cross combinations, e.g. CO and NCO hotspots

>>We cannot perform such a comparison of recombination hotspot positions as the precise recombination sites cannot be determined without whole genome sequences (we only have GBS data) and because of the low density of SNPs between parents.
