# Supplementary material for: Identification of quantitative trait loci (QTLs) for key cheese making phenotypes in the blue-cheese mold Penicillium roqueforti
Source: PLoS Genet. 2025 Apr 15;21(4):e1011669. doi: 10.1371/journal.pgen.1011669 (PMC12047768; doi:10.1371/journal.pgen.1011669)
Supplement: Attachment — Submitted filename: Review_&_response2.docx [file pgen.1011669.s024.docx]

Dear Editor,

We thank you and the referees again for the constructive and useful comments. We have revised the manuscript according to the suggestions. All comments were taken very seriously and you will find below specific answers to each one of the referees comments as well as your comments. In particular we have revised the narrow sense heritability using computations based on actual genetic relatedness, as suggested, and revised Introduction and Discussion sections, broadening the scope. In addition to addressing the referees’ comments, we have run the recent *Starfish* pipeline to gain more power in detecting *Starships*, and actually detected footprints of their presence in one translocation; we have added this information.

Changes are highlighted in yellow in the manuscript. We are sorry for the delay in resubmission, but we had issues submitting data to Genebank due to the hacking of our university that blocked all transmissions to Genebank. Note that we have changed the corresponding author as Ewen Crequer left the laboratory and did not have time for the resubmission.

We hope the revised version will now be satisfactory for publication in PLOS Genetics.

Best regards

Thibault Caron, on the behalf of all authors

Dear Dr Crequer,

Thank you very much for submitting your Research Article entitled 'Identification of quantitative trait loci (QTLs) for key cheese making phenotypes in the blue-cheese mold Penicillium roqueforti' to PLOS Genetics.

The manuscript was fully re-evaluated at the editorial level and by independent peer reviewers. Based on the reviews, we will not be able to accept this version of the manuscript, but we would be willing to review a much-revised version. We cannot, of course, promise publication at that time.

The following aspects must be fully solved in order for the manuscript to be acceptable for publication:

The results section related with heritability needs to be revised addressing adequately all the concerns and suggestions put forward by the Reviewers' and the Section Editor's comments.

>>We have changed the narrow-sense heritability computations as recommended.

In addition, the Discussion section should be significantly revised. The changes should aim to remove redundancy with the Results section, discuss the results in a broader context, and yield a concise and easily readable text for a non-specialist audience. Detailed suggestions are given in the Section Editor's comments below.

>>We have reduced the discussion section and added sentences and references to broaden the context.

Smaller issues that must also be addressed:

Lines 766 to 773 contain redundancies.

>> This part has been deleted.

Accession numbers should be provided with the submission.

>>We have added BioProject, BioSample and SRA accession numbers for all sequencing experiments reported here in supplementary table 5. Genome assemblies are currently in the processing status at GenBank.

Should you decide to revise the manuscript for further consideration here, please provide a detailed list of your responses to the review comments and a description of the changes you have made in the manuscript. Also, please note that this will be the last revision round. Should the changes introduced in the manuscript be deemed insufficient, the manuscript will not be further considered for publication at PLOS Genetics.

If you decide to revise the manuscript for further consideration at PLOS Genetics, please aim to resubmit within the next 60 days, unless it will take extra time to address the concerns of the reviewers, in which case we would appreciate an expected resubmission date by email to plosgenetics@plos.org.

You can use this link to log into the system when you are ready to submit a revised version, having first consulted our [Submission Checklist](http://c05y1x9s.r.us-east-2.awstrack.me/L0/http:%2F%2Fjournals.plos.org%2Fplosgenetics%2Fs%2Fsubmit-now%23loc-submission-checklist/1/010f019297a03301-691076b7-4f31-442b-a461-1f75e7b2811f-000000/jL66bxbdJLjAwLNbXqJUmaKSkHg=180).

To enhance the reproducibility of your results, we recommend that you deposit your laboratory protocols in protocols.io, where a protocol can be assigned its own identifier (DOI) such that it can be cited independently in the future. Additionally, PLOS ONE offers an option to publish peer-reviewed clinical study protocols. Read more information on sharing protocols at [https://plos.org/protocols?utm_medium=editorial-email&utm_source=authorletters&utm_campaign=protocols](https://c05y1x9s.r.us-east-2.awstrack.me/L0/https:%2F%2Fplos.org%2Fprotocols%3Futm_medium=editorial-email%26utm_source=authorletters%26utm_campaign=protocols/1/010f019297a03301-691076b7-4f31-442b-a461-1f75e7b2811f-000000/0nhvUTaEw7UGItFmMugiWepB_gM=180)

Please be aware that our [data availability policy](http://c05y1x9s.r.us-east-2.awstrack.me/L0/http:%2F%2Fjournals.plos.org%2Fplosgenetics%2Fs%2Fdata-availability/1/010f019297a03301-691076b7-4f31-442b-a461-1f75e7b2811f-000000/una_VnxFao5etqYSrkIZKALrSZc=180) requires that all numerical data underlying graphs or summary statistics are included with the submission, and you will need to provide this upon resubmission if not already present. In addition, we do not permit the inclusion of phrases such as "data not shown" or "unpublished results" in manuscripts. All points should be backed up by data provided with the submission.

While revising your submission, please upload your figure files to the [Preflight Analysis and Conversion Engine](http://c05y1x9s.r.us-east-2.awstrack.me/L0/http:%2F%2Fpace.apexcovantage.com%2F/1/010f019297a03301-691076b7-4f31-442b-a461-1f75e7b2811f-000000/leqJ9L6rIoRn53bfjgMREain4CM=180) (PACE) digital diagnostic tool. PACE helps ensure that figures meet PLOS requirements. To use PACE, you must first register as a user. Then, login and navigate to the UPLOAD tab, where you will find detailed instructions on how to use the tool. If you encounter any issues or have any questions when using PACE, please email us at figures@plos.org.

PLOS has incorporated [Similarity Check](http://c05y1x9s.r.us-east-2.awstrack.me/L0/http:%2F%2Fwww.crossref.org%2Fcrosscheck.html/1/010f019297a03301-691076b7-4f31-442b-a461-1f75e7b2811f-000000/uJe8OuvMTzVTnfT4YXq2rbifV9o=180), powered by iThenticate, into its journal-wide submission system in order to screen submitted content for originality before publication. Each PLOS journal undertakes screening on a proportion of submitted articles. You will be contacted if needed following the screening process.

To resubmit, log into your Editorial Manager account and select the option 'Revise Submission' in the 'Submissions Needing Revision' folder.

We are sorry that we cannot be more positive about your manuscript at this stage. Please do not hesitate to contact us if you have any concerns or questions.

Yours sincerely,

Paula Gonçalves
Guest Editor
PLOS Genetics

Kelly Dyer
Section Editor
PLOS Genetics

Justin Fay
Section Editor
PLOS Genetics

**Section Editor's comments and suggestions:**

1) The heritability section needs revision. Heritability based on parent-offspring correlations is not reliable with a small number of families. Falconer and Mackay suggest >100. The manuscript has 5 crosses (families) and correlations based on 5 points is expected to be quite noisy. Broad and narrow sense heritability should be estimated using standard approaches applicable to cross: PMC4001867. The authors estimate Broad-sense heritability from repeatability of trait measurements and Narrow-sense heritability by comparing phenotypic similarity among individuals with their actual genetic relatedness. It seems like the authors have the data to do both of these.

>>We thank the editor very much for this insightful suggestion. We have deleted the computations of heritability that were made based on the correlation across the five crosses and used instead the approach applied in Bloom et al. (2013) to compute narrow-sense heritability values L293-306. This has not changed the conclusions.

3) The introduction is ok (but could be better), but discussion should really put the work into the context of a larger body of literature in order to be relevant/interesting to a broad audience. I think the paragraph on crossovers is quite nice/concise, but the other ones are mainly reiterating results and only put into the context of cheese literature.

>> The introduction has been modified as suggested. We also eliminated the redundancy in the discussion and broadened the context, including references to previous work on yeasts and domesticated plants.

Transgressive segregation: how do these results compare to others in the field (not just cheese studies), this is important for broader audience interpretation of this result. A comprehensive review is not required but there are certainly other fungal studies that have examined this issue.

>> We have added sentences and references for other fungi L424-429.

Translocations and segregation biases: Are translocations commonly found in crosses of domesticated (with wild or other domesticated) organisms? Is this result expected or not and what is the implication for domestication/improvement. The context of this result is well described in relation to P. roqueforti but not other domesticated organisms.

>> We have added sentences and references for other organisms L448-455.

Crossing over by chromosome and recombination rate: This section is concise and draws nice only the broader context of the result in comparison to prior studies.

QTL identification and the genomic architecture of adaptation: ok

Strain breeding in Penicillium roqueforti: quite specialized discussion should be broader or much more concise

>> This part has been deleted.

**Reviewer's Responses to Questions**

**Comments to the Authors:
Please note here if the review is uploaded as an attachment.**

Reviewer #1: I'm still confused the the narrow-sense heritability estimates. There are still negative values and two values greater than 100% (Supplementary Table 3). How could these be? By definition, narrow-sense heritability (or any heritability estimate) should be a value between 0% (when no genetic variation contributes to observed phenotypic variation) and 100% (when all the observed phenotypic variation could be explained by allelic differences).

>> We have deleted the computations based on correlations across the five crosses, and have added new computations based on the actual genetic relatedness between individuals, as recommended by the editor (L293-306).

Lines 770-773 are redundant.

>>We have deleted this part.

Reviewer #2: The revised version is generally unsatisfactory. In fact, they don't answer certain questions and try to avoid solving problems.

1. Concerning genetic diversity, the author writes in the abstract that "the two populations of domesticated cheeses present very little genetic diversity", then in the author summary that "the two populations of domesticated cheeses of P. roqueforti each present very little genetic diversity…”. And then they still have an introduction of more than 3 pages and we still do not have the values of this diversity between these two populations. And what is the estimated divergence between the parents of the generated map population?

>> We have added the nucleotide diversity values previously computed for each population in the introduction (L127-130). We have also estimated the divergence between the parental strains using the average nucleotide identity and added corresponding explanations in M&M (L620-626), and values in the result section (L168-169) and in Table 1.

2. The introduction is still too long and it will really have to be shortened to get straight to the point. Concerning the discussion (5 pages in total), it's the same. The discussion remains essentially redundant with the results section. As it stands, it's really unpleasant to read. It's really too wordy for not much.

>>We have modified the introduction and removed redundancy in the discussion. Moreover, following the editor’s comment, we have broadened the context of the discussion.

3. The point raised by the reviewer 1 regarding the heritability is interesting. It is in fact highly problematic and was not solved in the new version. It is also even mentioned in the responses of the authors: 'However, we agree that our narrow sense values are in some cases problematic but what causes it is more likely segregation bias which will favor one parental allele over the other, thus modifying the average phenotypic value of the offspring’. But again, they don’t propose to solve that. In addition, they wrote in the main text ' Some estimates were below zero or higher than 100% (Figure 4), but heritability estimates based on regressions between offspring and parent trait means may not be highly reliable (Steinsaltz et al., 2020)’. If not highly reliable, why using them? Not sure to understand how they deal with that - not reliable but not a big deal?

>> We apologize for the lack of clarity in our previous justification. We have removed the estimates of narrow-sense heritability based on correlations and added new computations based on genetic relatedness between individuals.

Reviewer #3: I don't have further feedback for the authors.

**Have all data underlying the figures and results presented in the manuscript been provided?**
Large-scale datasets should be made available via a public repository as described in the *PLOS Genetics* [data availability policy](http://c05y1x9s.r.us-east-2.awstrack.me/L0/http:%2F%2Fjournals.plos.org%2Fplosgenetics%2Fs%2Fdata-availability/2/010f019297a03301-691076b7-4f31-442b-a461-1f75e7b2811f-000000/FqMLokfLAYjJzDL09PGe8Ps1zfo=180), and numerical data that underlies graphs or summary statistics should be provided in spreadsheet form as supporting information.

Reviewer #1: **No:** Most genomic and raw phenotypic data aren't presented/available. They should be publicly accessible before formal acceptance and publication.

>>All genomic and raw phenotypic data have been made available, see genome accession numbers in Supplementary Table 5 and Supplementary Materials.

Reviewer #2: Yes

Reviewer #3: None

PLOS authors have the option to publish the peer review history of their article ([what does this mean?](https://c05y1x9s.r.us-east-2.awstrack.me/L0/https:%2F%2Fjournals.plos.org%2Fplosgenetics%2Fs%2Feditorial-and-peer-review-process%23loc-peer-review-history/1/010f019297a03301-691076b7-4f31-442b-a461-1f75e7b2811f-000000/9B5UFhEdO59bW8GcBh5DRX_kEHQ=180)). If published, this will include your full peer review and any attached files.


If you choose “no”, your identity will remain anonymous but your review may still be made public.


**Do you want your identity to be public for this peer review?** For information about this choice, including consent withdrawal, please see our [Privacy Policy](https://c05y1x9s.r.us-east-2.awstrack.me/L0/https:%2F%2Fwww.plos.org%2Fprivacy-policy/1/010f019297a03301-691076b7-4f31-442b-a461-1f75e7b2811f-000000/e9Ousdn2Z3uBbL2SlTz5eRAMpMA=180).

Reviewer #1: No

Reviewer #2: No

Reviewer #3: No
